# Screening of potential immune-related genes expressed during sepsis using gene sequencing technology

**DOI:** 10.1038/s41598-022-23062-7

**Published:** 2023-03-14

**Authors:** Ye Tian, Chenglin Wang, Qiangyong Lu, Chuan Zhang, Lin Hu, Jiamei Ling, Muhu Chen, Yingchun Hu

**Affiliations:** 1 Department of Emergency Medicine, Affiliated Hospital of Southwest Medical University, Luzhou, 646000 Sichuan China; 2 Department of Pediatrics, Ya’an Second People’s Hospital, Ya’an, China; 3 Internal Medicine, Affiliated Hospital of Ya’an Vocational and Technical School, Ya’an, China; 4 Department of Pediatrics, Lushan County People’s Hospital, Ya’an, China; 5 Department of Obstetrics and Gynecology, Lushan County People’s Hospital, Ya’an, China

**Keywords:** Diseases, Pathogenesis

## Abstract

To screen potential pivotal targets in sepsis through peripheral blood. Septic patients (n = 23) and healthy volunteers (n = 10) were enrolled according to SEPSIS 3.0. Peripheral blood was collected within 24 h of enrollment, RNA-seq was performed on the peripheral blood. The sequencing data was screened for DEGs (*p* < 0.01; logFC ≥ 2). PPI, WGCNA and survival curve analysis were used to identify potential targets. Then, 5 PBMC samples were conducted by single-cell sequencing for cell lineage location. Finally, mouse sepsis model and clinic samples were performed to verify the targets gene using RNA-seq and RT-PCR, respectively. Compared to the control group, 1007 DEGs were found in septic group. BCL9L, BCL11B, CD247, CD96, MAFG and SAMD3 were in the core of network. These six genes correlated to the survival rate of septic patients and they were mainly expressed in T cells, except that MAFG was located in monocyte cell. The expression levels of six key genes were confirmed by animal and clinical samples. BCL9L, BCL11B, CD247, CD96 and SAMD3 were decreased in sepsis and mainly expressed in the T cell; while MAFG increased in sepsis and localizes to monocytes. These genes may be therapeutic targets for sepsis.

## Introduction

Sepsis is a significant cause of death in the ICU. Currently, strategies to treat sepsis include antibiotics, intravenous infusion, and pressor agents. However, therapeutic options remain limited. Therefore, it is critical to understand the pathogenesis and pathophysiology of sepsis to develop new therapeutic targets^1,2^. Sepsis is a clinical disease caused by infection with a high inflammatory response (i.e., the abnormal early activation of innate immunocytes, including macrophages and neutrophils). Patients will then exhibit tolerance or immune paralysis^3,4^, causing a decrease in lymphocytes and dysfunction of immune cells^5^. It is recognized that sepsis-induced white cell dysfunction and immunosuppression are essential factors that cause high morbidity and mortality^1^.

The present methods to diagnose and treat sepsis are not adequately specific, sensitive, and fast^6,7^. Therefore, it is critical to identify new diagnostic and therapeutic targets. RNA-sequencing is an emergent technology to identify and quantity RNA molecules in biological samples^8^. Differentially expressed genes can be obtained between two or more cell populations through differentially expressed gene (DEG) analysis; RNA-seq cannot identify differentially expressed genes among cells because of the cell mixture used. The presentation of single-cell RNA-seq (scRNA-seq) further promotes the development of this field. The technology of scRNA-seq can detect gene expression in every single-cell. This provides a more general and comprehensive viewpoint on how cells coordinate to make specific reactions^9,10^.

The present study aims to demonstrate key targets’ expression profiles and cell identity using dimensional sequencing technologies. Differentially expressed genes were found and screened by RNA-seq technology and bioinformatical analysis. Cellular identity expressing core genes were specified using 10 × single-cell sequencing. Then, the sequencing of peripheral blood samples from LPS-induced sepsis mice was applied to verify the cells that express these potential target genes, which provides a basis for further in vivo functional studies.

## Methods

### Clinical sample collection

Human blood samples were collected from septic patients (n = 23) within 24 h of enrollment in the ICU/EICU of Southwest Medical University-affiliated hospital between Jan. 2019 and Dec. 2020. Blood samples were collected from healthy volunteers as a control (n = 10). According to the company's instructions, the PAXgene system was used to collect peripheral blood samples. Blood samples were stored at − 80 °C in the biological sample bank of Southwest Medical University-affiliated hospital. Enrolled patients were diagnosed according to the SEPSIS 3.0 criteria (i.e., evidence of infection with more than two organ dysfunction). Excluding criteria involved (1) age less than 16 or more than 65, (2) history of defined organ function failure, (3) history of HIV or leukemia; (4) patients or families unwilling to participate. This study was approved by the ethical commission of Southwest Medical University-affiliated hospital (No. ky2018029) with clinical trial No. ChiCTR1900021261. Before the trial, all enrolled patients or their families signed consent forms to ensure that they were informed. Participants in current study have been performed in accordance with the Declaration of Helsinki.

### RNA-sequencing

Total RNA was extracted from blood samples using Trizol, Agilent 2100 (Thermo Fisher Scientific, MA, USA) for quantification analysis. First, ribosomal RNA was removed using enzyme H reagent targeting specific oligonucleotides and ribosides. After purification, the RNA was fragmented into small pieces using SPRI beads by bivalent cation under high temperatures. Reverse transcriptase and randomized primers were used to copy the RNA fragments into the first strand of cDNA. Then, the second strand of cDNA was synthesized using DNA polymerase I and RNase H. Agilent 2100 bioanalyzer was used to measure the fragment size distribution. Quantification of the library pool was performed using qPCR analysis. According to the manufacturer’s protocol, the BGISEQ-500/MGISEQ-2000 system (BGI-Shenzhen, China) was then used to make a sequencing analysis of the eligible library pool. The obtained reads were removed the adapters, low-quality reads and reads with unknown base N content greater than 5%. We define reads whose quality value is less than 10 and whose proportion of bases in the total base number of the reads is more than 20% as low-quality reads. The clean reads were filtered by SOAPnuke software to guarantee reliability of the results and were saved into FASTQ format. The clean reads were aligned with the reference genome using HISAT and bowtie2 software. The datasets generated during the current study are available in the CNGBdb repository, (https://db.cngb.org/, project No. CNP0002611).

### Differentially expressed gene screening

An online analysis platform (https://www.xiantao.love/) based on R (version 3.6.3) was used to perform log2 normalization on the expression matrix. DESeq2^11^ and ggplot2 package were used for statistical analysis and visualization of the data, respectively. To rationally screen the differentially expressed genes(DEGs), the differentially expressed threshold parameters were set as *p* < 0.01 and log2FC ≥ 2.

### Gene ontology (GO) functional enrichment analysis

GO is a method to make a categorical description of biological processes (BP), cellular components (CC), and molecular functions (MF)^12^. This study focused on immune responses and functional alterations of immunocytes. The clusterProfile package^13^ was used to perform GO analysis further to explore the overall functional enrichment of DEGs. *p* < 0.05 was considered statistical significance. Gene Set Enrichment Analysis (GSEA) is an approach to determine the contribution to phenotypes based on the distribution tendency of ranked relevant phenotypes. It aids in determining the enrichment tendency of upregulation or downregulation of differentially expressed genes. Significant enrichment thresholds were set as FDR < 0.25 and an adjusted *p* < 0.05.

### PPI and WGCNA screening for pivotal genes

Protein–protein interaction (PPI) is widely used to screen critical genes. The principle of this method is based on the established connection network according to evidence strength of the interaction between two previously found proteins. Theoretically, the closer a protein to the central area with more external connection, the more potential of this protein to be a key target. To further screen potential immune-related key genes, STRING11.5 (https://cn.string-db.org/) was used to construct a PPI network of relevant genes, which related to immune response, leukocyte mediated immunity, intercellular communication, cell secretion etc*.* It facilitates screening genes in the center of the network to further reduce the range of critical genes. The present study’s connection strength between the two factors was set as no less than 0.4.

Co-expression analysis is another important method of core gene screening, which is based on the principle of correlation between gene expression values and clinical manifestations. The online analysis platform iDEP (http://bioinformatics.sdstate.edu/idep/) was adopted for co-expression analysis.According to the software flow, we chose the soft threshold of 8, and set a module to require more than 20 genes. Those modules related to clinical phenomena were selected, and hub genes were to construct the network based on the correlation coefficient between any two genes greater than 0.4.

### Survival curve of pivotal genes

Survival curve analysis has important clinical significance in analyzing the correlation between critical genes identified by screening and clinical characteristics. To explore if target genes identified by the PPI/WGCNA method have the potential to determine prognosis, the GSE65682^14^ data set, along with clinical features from the GEO database, were downloaded and analyzed. This data set includes peripheral blood gene expression data from approximately 400 septic patients, with their corresponding survival information within 28 days. The core genes selected above were ranked from low to high expression values, and took the median value as the boundary. The first half was named as the low expression group (n = 239), while the latter half was defined as high expression group (n = 239). GraphPad Prism (version 7.0, https://www.graphpad-prism.cn/) software was used to plot and analyze the relevant data extracted. Log-rank test was used for statistical analysis. *p* < 0.05 was considered statistically significant.

### Single-cell sequencing

Peripheral blood cells are a mixture of multiple cell populations. Single-cell sequencing analysis helps determine the cellular location of the target genes in tissues. In the present study, 10 × single-cell sequencing technology was applied to explore the location of each target gene in cell lineages. This method is performed according to the manufacturer’s protocol. Five blood samples (NC = 2; SIRS = 1; SEPSIS = 2) were collected and mixed. Raw reads from high-throughput sequencing were in FASTQ format, and sequences were subjected to 10 × genomics software CellRanger for quality analysis. The Seurat software package^15^ was used to further quality control the data. Gene expression was used for PCA dimension reduction, followed by visualization of results through tSNE. In addition, the FindAMarkers function was used to identify gene biomarkers. The identified genes were visualized through the VlnPlot and FeaturePlot functions. The specification of cell location of specific target genes facilitated the selection of specific cell lineages for the following in vitro function study.

### Animal model establishment and sequencing verification

To verify the expression of the genes selected above in mouse peripheral blood, we established an LPS-induced sepsis model in mice, a previously reported method^16^. Sepsis l was induced with administration of 30 mg/kg LPS by tail vein injection. An equivalent volume of saline was given to the control group. Mouse peripheral blood was collected 72 h post-LPS administration and was subjected to gene sequencing analysis. Finally, the expression data of the target genes identified from sepsis patients were extracted for statistical analysis, and a *p* value of less than 0.05 was considered a statistical difference. Animal experiment was approved by the ethical commission of Southwest Medical University (No. ky20180300268). All methods were applied in accordance with ARRIVE guidelines. All methods were carried out in accordance with relevant guidelines and regulations.

### RT-qPCR validation for clinical samples

The validation of clinical samples is more conducive to the reliability of the core gene expression trend. RNA samples of clinical were extracted according to the company's operating instructions (PAXgene blood RNA Kit: HY-13221). RNA purity meet the following conditions: 1.8 < A260/A280 < 2.0. Total RNA was reverse transcribed into cDNA according to the instructions (PrimeScript cDNA Synthesis Kit, Takara). PCR amplification conditions were initial denaturation at 94 °C for 3 min followed by 40 cycles at 94 °C for 5 s, the annealing and extension at 60 °C for 30 s. Primer sequences of key genes were as follows: GAPDH-F: CAATGACCCCTTCATTGAC, GAPDH-R: CGCTCCTGGAAGATGGTGA(141 bp); BCL11B-F:GGTGCCTGCTATGACAA,GGCTCGGACACTTTCCTGAG(80 bp); BCL9L-F:TCTCGCC TAGCAACTCAAGT, BCL9L-R:GAGCACCATTCGTCCCCAC(226 bp); CD247F:GGCACAG TTGCCGATTACAGA,CD247-R:CTGCTGAACTTCACTCTCAGG(132 bp);CD96-F: CAAAC ACAGACAGTAGGCTTCT, CD96-R:GGGGATGATAGACAGCAATCA(85 bp); MAFG-F: TCAGATTTCAGAGGAATACCCAGCAG, MAFG-R: TGATCACCAGTCAGAAGTGTAC ACAC(149 bp); SAMD3-F:TGGTCAGTTGAGCAGGTCT, SAMD3R:GGCCCCACTTACTT CTTCCT(90 bp). In this study, GAPDH was used as an housekeeping gene, and the relative expression of key genes was calculated by 2^−△△CT^.

### Statistical analysis

Raw data from RNA-sequencing was compared after log2 transformation. Each group’s common measurement data were presented as mean ± SD, followed by a t-test. *p* < 0.05 was considered statistically significant.

## Results

### Clinical information on septic patients

**Figure 1:**
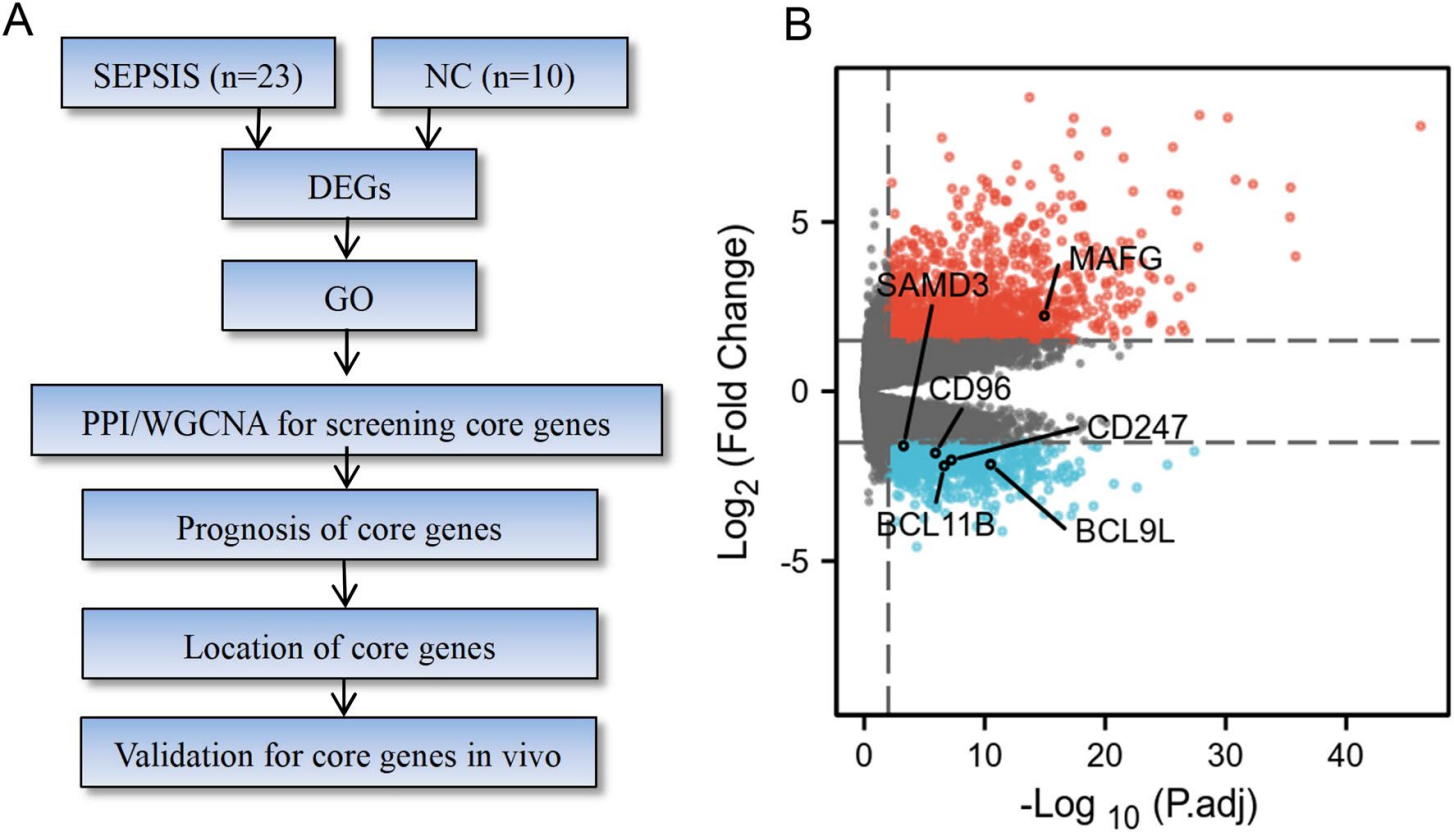
Study workflow and screening of differentially expressed genes. (**A**): Workflow of this study. Firstly, human blood samples were collected from septic patients and healthy volunteers. RNA-seq technology and a bioinformatical approach were used for screening differentially expressed genes. Secondly, PPI analysis combined with clinical characteristics was used to make the final definition of 6 potential pivotal target genes. Lastly, single-cell sequencing was used to identify the cell lineage location of target genes. And peripheral blood sequencing from the septic mouse model was used to verify pivotal the expression profiles of key genes. (**B**): Volcano plot to screen differentially expressed genes. Horizontal ordinate presented as log_2_ (FC), each dot indicating a gene. Red color as upregulation; Blue color as downregulation. CD247, BCL9L, CD96, SAMD3 and BCL11B were downregulated in the septic group, while MAFG were up-regulated in the septic group.

A total of 23 septic patients and 10 healthy volunteers were included in the study. The workflow of this study is shown in Fig. 1A. According to SEPSIS 3.0 criteria, all septic patients should suffer from more than two organ functional injuries. In this study collected patients’ gender, age, WBC, DBILI, creatinine, and hemagglutination relating to clinical organ dysfunction, as shown in Table 1. It was demonstrated in the septic group that indexes of inflammatory and organ functional damage significantly increased, accompanied by two or more organ dysfunction.

**Table 1:** Clinical Characteristics of all subjects.

Clinical variable	Normal control (n = 10)	Septic patient (n = 23)	*p* value
Age (years)	51.1 ± 2.648	55.38 ± 2.38	0.38
Sex (male), n (%)	6(60%)	27(73%)	0.46
DBILI (umol/L)	4.14 ± 0.619	11.39 ± 1.512	0.0179
SCr. (umol/L)	52.32 ± 4.065	158.6 ± 26.2	0.0424
WBC (10^9/L^)	5.797 ± 0.303	10.93 ± 1.096	0.0202
PT (s)	10.68 ± 0.2004	18.8 ± 1.913	0.034
Fib (g/L)	2.803 ± 0.1743	5.124 ± 0.4108	0.0058
Bacteria and fungi culture			
Positive rate, n (%)		11(47.8%)	
G^+^, n (%)		3(13.0%)	
G^−^, n (%)		4(17.4%)	
G^+^ and G^−^, n (%)		5(21.7%)	

### Differentially expressed gene and function enrichment

**Figure 2:**
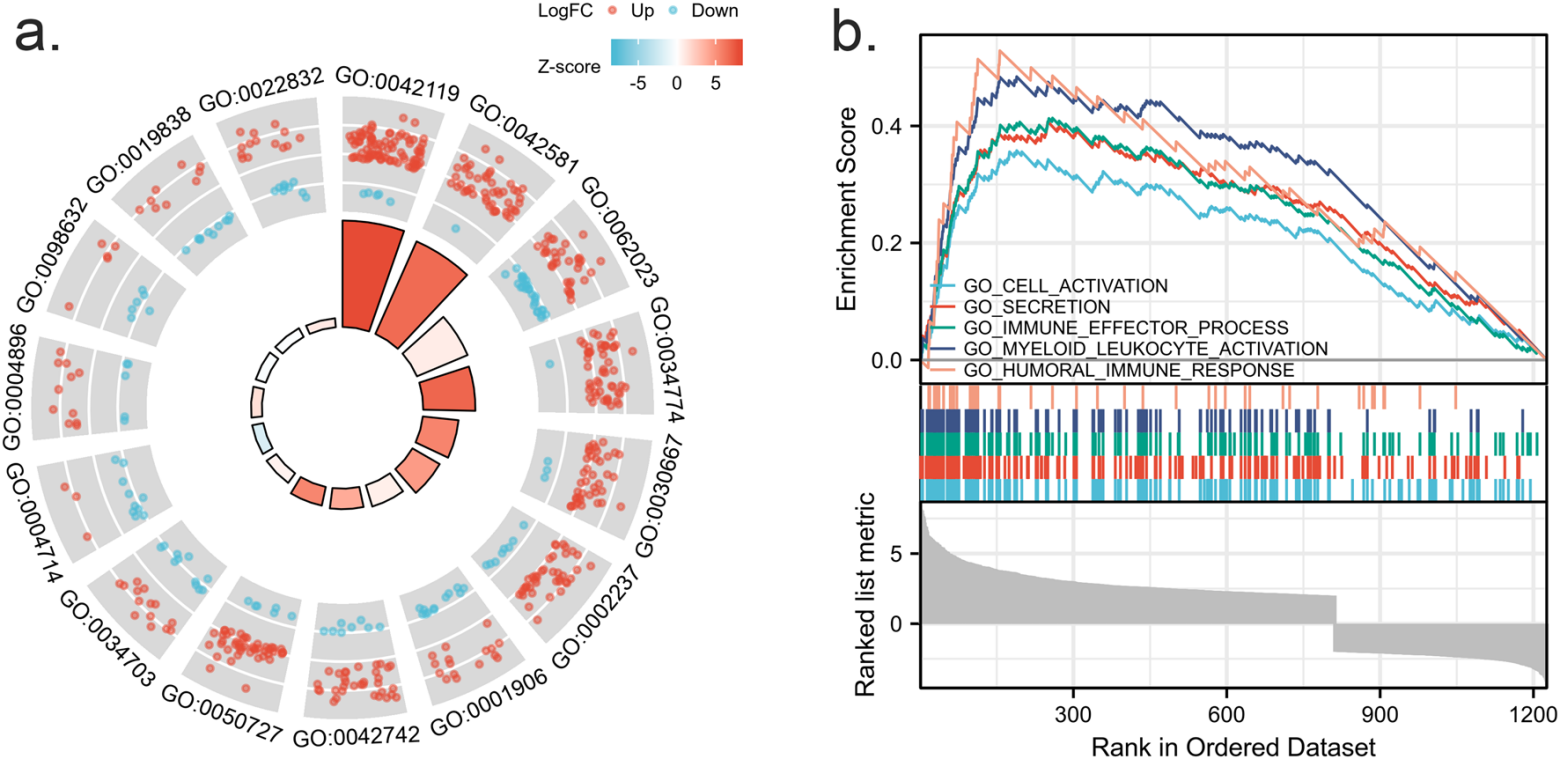
GO enrichment analysis of differentially expressed genes. (**A**): Function enrichment circle plot of top5 enriched GO terms from BP, MF and CC analysis. Red color as upregulated genes; Blue color as downregulated genes, with each dot representing a gene. The height of the column in the middle indicated the number of enriched genes. (**B**): GSEA functional analysis plot of immune-related genes. Genes with functions relating to cell activation, secretion, immune effector process, myeloid leukocyte activation, and humoral immune response are predominantly enriched in the front half section, suggesting an improved function in sepsis.

A total of 1007 differentially expressed genes were discovered from the two groups of peripheral blood after normalization. Compared to the control group, 660 genes were upregulated, and 347 were downregulated in the septic group (Fig. 1B). All DEGs can be seen in "Appendix" (List of differentially expressed genes). GO function enrichment analysis showed that these differentially expressed genes were associated with host anti-pathogen BP, including neutrophil activation, response to molecule of bacterial origin, cell killing, defense response to the bacterium, and regulation of inflammatory response. Cell content distribution (CC) showed that these differentially expressed genes related to cellular exocrinosity, for example, specific granule, collagen-containing extracellular matrix, secretory granule lumen, secretory granule membrane, and cation channel complex. The MF of these differentially expressed genes was mainly enriched in the processes of transmembrane receptor protein tyrosine kinase activity, cytokine receptor activity, cell–cell adhesion mediator activity, growth factor binding, and voltage-gated channel activity (Fig. 2A), as shown in Table 2. GSEA ranking analysis indicated the upregulation of cell activation, secretion, immune effector process, myeloid leukocyte activation, and humoral immune response in the septic group (Fig. 2B). These findings suggested an improved inflammatory response in immunocytes and enhanced cell activation during sepsis.

**Table 2:** Go enrichment of DEGs.

Terms	ID	Description	Count	*p*. adjust	Q value
BP	GO:0,042,119	Neutrophil activation	91	1.2576E−24	1.01587E−24
BP	GO:0,002,237	Response to molecule of bacterial origin	48	7.2361E−08	5.84522E−08
BP	GO:0,001,906	Cell killing	28	8.3204E−06	6.72107E−06
BP	GO:0,042,742	Defense response to bacterium	41	2.045E−05	1.65188E−05
BP	GO:0,050,727	Regulation of inflammatory response	52	4.2417E−05	3.42637E−05
CC	GO:0,042,581	specific granule	49	1.7415E−23	1.46014E−23
CC	GO:0,062,023	Collagen-containing extracellular matrix	61	4.0492E−13	3.39495E−13
CC	GO:0,034,774	Secretory granule lumen	52	1.7265E−12	1.44758E−12
CC	GO:0,030,667	Secretory granule membrane	43	7.2171E−09	6.05108E−09
CC	GO:0,034,703	Cation channel complex	25	0.00175885	0.001474677
MF	GO:0,004,714	Transmembrane receptor protein tyrosine kinase activity	13	0.00230035	0.002026902
MF	GO:0,004,896	Cytokine receptor activity	16	0.00289464	0.002550552
MF	GO:0,098,632	Cell–cell adhesion mediator activity	11	0.0033865	0.002983942
MF	GO:0,019,838	Growth factor binding	19	0.00473805	0.004174826
MF	GO:0,022,832	Voltage-gated channel activity	23	0.00777128	0.006847495

### PPI and WGCNA* screening of pivotal genes*

**Figure 3:**
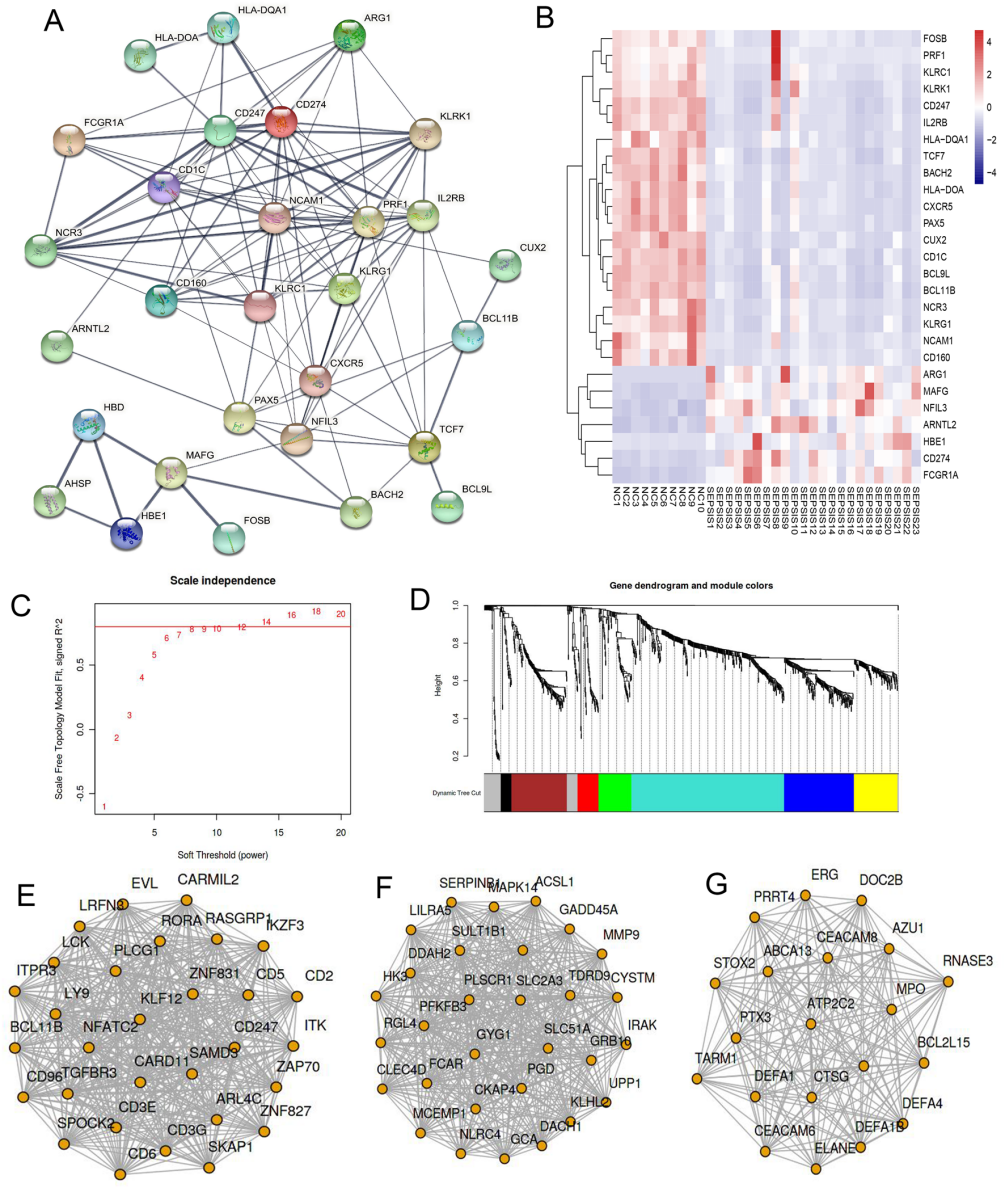
PPI and WGCNA plot and heatmap of pivotal gene expression. (**A**): PPI module plot of immune-related key genes. PPI analysis showed widely connection of immune-related genes, among which CD160, CD247, BCL9L, and BCL11B are located in the center of the network. (**B**): Heatmap of pivotal gene expression in PPI modules. Up-regulated genes are labeled as red; down-regulated genes are labeled as blue, and sample number at the bottom, gene names at right, and the shades of color indicating expression levels. (**C**): The soft threshold was 8 by WGCNA screening. (**D**): Visualization of cluster analysis of different modules.The blue, yellow and green modules were consistent with the clinical presentation. (**E**–**G**): Co-expression network of potential core genes in module 2 (blue), module 4 (yellow) and module 5 (green), respectively.

Based on the identification of the gene set enriched in immune response, more than 20 genes in the center area of the PPI network, including IL2RB, CD247, CD1C, CD160, BCL9L, BCL11B, TCF7, and MAFG, by PPI analysis, function enrichment analysis showed that these factors were associated with inflammatory response, secretory factors, and cellular communication (Fig. 3A). Among these factors, CD247, PAX5, BCL9L, BCL11B, CD160, and IL2RB were decreased in the septic group. In contrast, ARG1, MAFG, NFIL3, CD274, and FCGR1A were increased in the septic group (Fig. 3B). According to the results of co expression analysis (Fig. 3C,D), three expression modules were identified, which were module 2 (Fig. 3E), module 4 (Fig. 3F), and module 5 (Fig. 3G). The gene expression in module 2 was decreased in sepsis, while the gene expression in modules 4 and 5 was high in sepsis. From the results, CD247, KLF12, BCL11B, ZAP70, CD3E etc. were located in the core of the co-expression network of module 2. GYG1, MMP9, FCAR, MAPK14 etc*.*were located in the central part of module 4, while MPO, BCL2L15, DEFA4, CEACAM6, RNAS3, CEACAM8 etc*.* were located in the core part of module 5.

### Association between pivotal genes and prognosis

**Figure 4:**
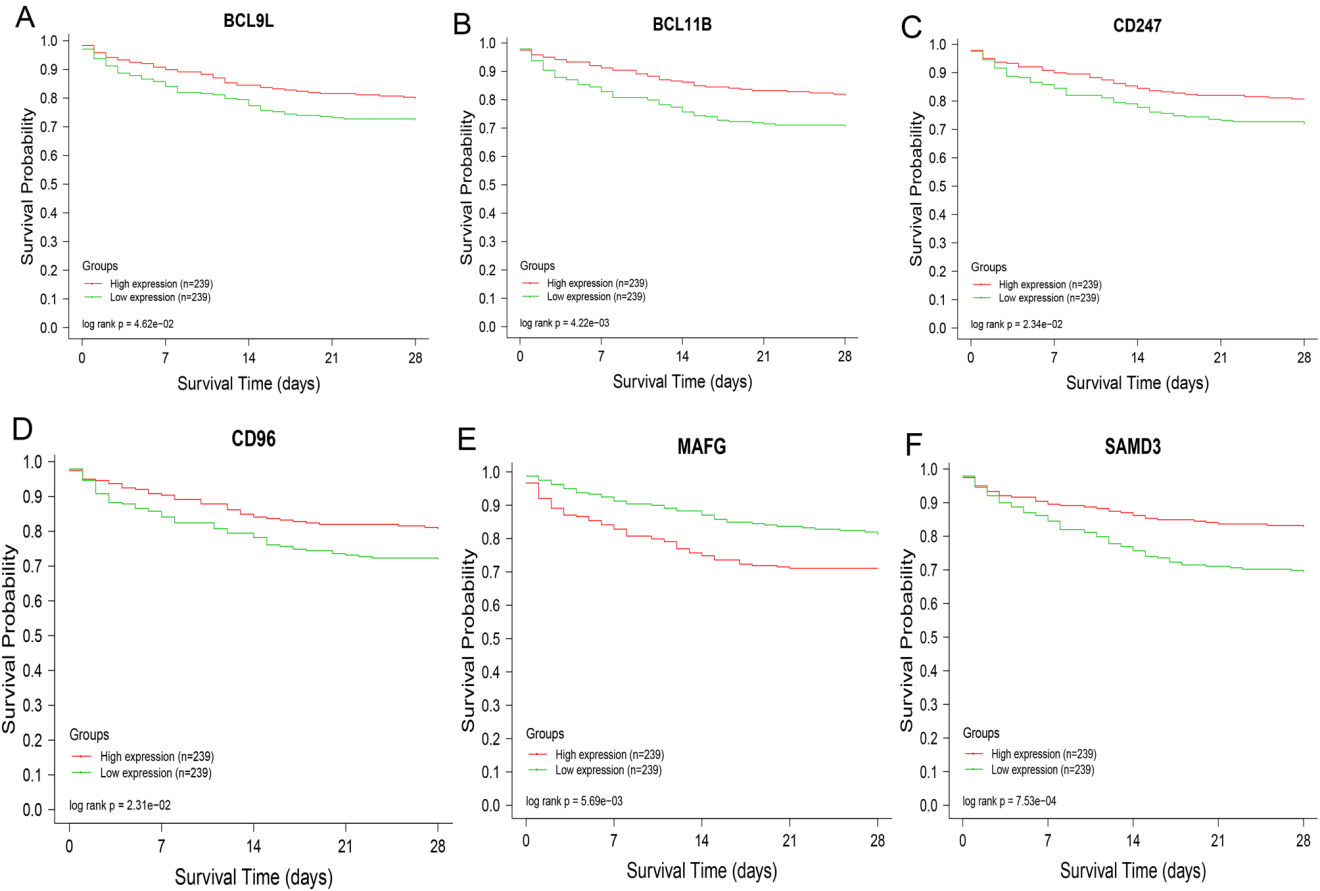
Survival curve of key genes. a-f showed 28-day temporary survival curves of BCL9L, BCL11B, CD247, CD96, MAFG and SAMD3, respectively (according to GSE65682), suggesting a positive correlation between these 5 pivotal genes and the survival rate of sepsis, except that MAFG showed negative correlation with survival rate. Red color as a high expressed group; Green color as a low expressed group. Horizontal ordinate as timepoints within 28 days; Vertical ordinate as survival rate. Log-rank statistical analysis showed that P_BCL9L_ = 0.0462, P_BCL11B_ = 0.00422, P_CD247_ = 0.0234, P_CD96_ = 0.0231, P_MAFG_ = 0.00569 and P_SAMD3_ = 0.00075.

Through analysis of clinical data on the prognosis of septic patients, it was found that patients with high levels of BCL9L, BCL11B, CD96, SAMD3 and CD247 tended to have relatively high survival rates (Log-rank *p* < 0.05, Fig. 4A–D,F), suggesting a positive correlation between these five genes and prognosis. MAFG showed the expression value was negatively correlated with the survival rate (Fig. 4E).This finding demonstrated that these genes are potential targets for research and therapy. According to the result of the survival curve, six potential pivotal targets were screened for the following verification analysis.

### Single-cell lineage location of pivotal genes

**Figure 5:**
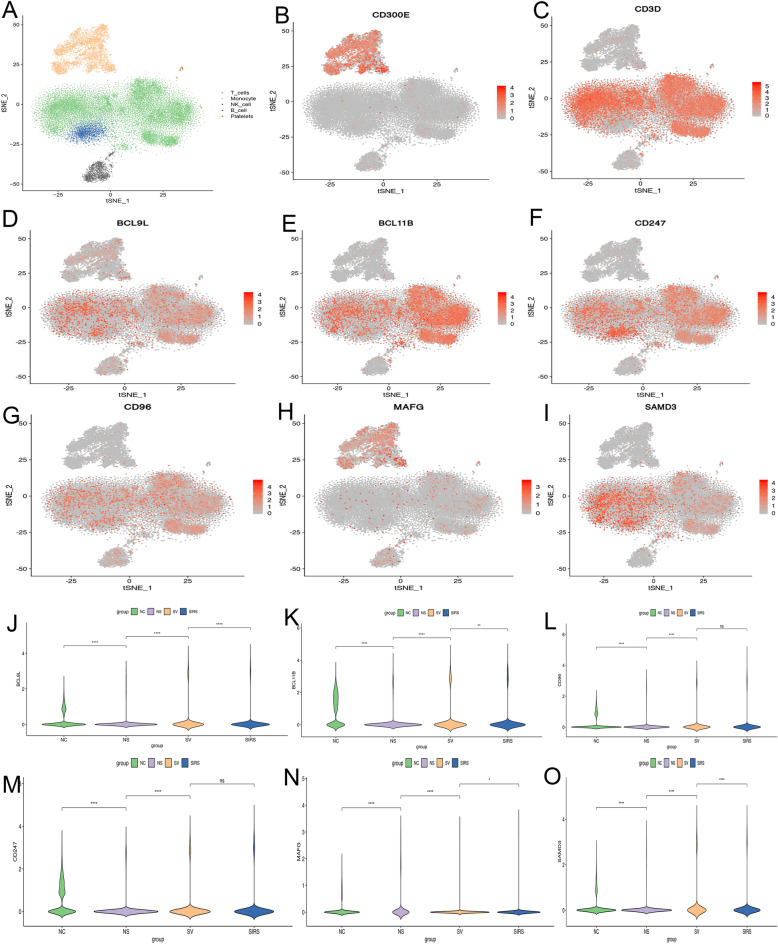
tSNE cell lineage location plot and multi-group comparison of pivotal genes. (**A**): tSNE overall plot of mixed samples from sepsis. Green color indicates T cell lineage; Yellow color indicates mononuclear-macrophage cell linage; Blue color indicates NK cell lineage; Grey color indicates B cell lineage. (**B**): CD300E as a biomarker for myeloid cells (positive control). (**C**): CD3D as a biomarker for T cells (positive control). (**D**–**I**) showed tSNE plots of BCL9L, BCL11B, CD247, CD96, MAFG and SAMD3, respectively. It suggesting these 5 essential genes were predominantly in the T cell lineage of PBMC from sepsis, only MAFG was located in monocyte cell lines. (**J**–**O**) showed violin plots of BCL9L, BCL11B, CD247, CD96, MAFG and SAMD3 expression in different samples. Vertical ordinate indicated relative expression levels. The abscissa represents different sample: NC for normal control 1; NS for non-survival sample of sepsis; SIRS for systemic inflammatory response syndrome; SV for survival sample of sepsis. ****: *p* < 0.0001; ***: *p* < 0.001; **: *p* < 0.01; *: *p* < 0.05; ns: *p* ≥ 0.05.

PBMC populations were categorized through PCA dimension reduction analysis. As identified by common markers, cell populations includes T cell lineages, NK cells, monocytes, B cells, and platelets (Fig. 5A). CD300E is a commonly used biomarker for monocytes (Fig. 5B). CD3D is used as an NK-T cell biomarker (Fig. 5c). Single-cell sequencing analysis showed that BCL9L, BCL11B, CD96, SAMD3and CD247 are mainly expressed in T cell lineages, while MAFG localizes to monocyte cell lines (Fig. 5D–I). These findings illustrated the cellular location of key genes, providing clues to future cell lineage selection for in vitro functional assay. The expression results of core genes in different samples showed that five genes (BCL9L, BCL11B, CD96, SAMD3 and CD247) were decreased in sepsis samples, compared with the normal group. MAFG was increased in sepsis samples, compared with the normal group. This was consistent with our previous sequencing data. Moreover, MAFG was increased in non-survivor samples, compare to survival (Fig. 5J–O). The expression values of five genes in survival samples were higher than those in death samples, while MAFG showed the opposite trend. It is consistent with the survival curve conclusion above.

### Verification of key genes

**Figure 6:**
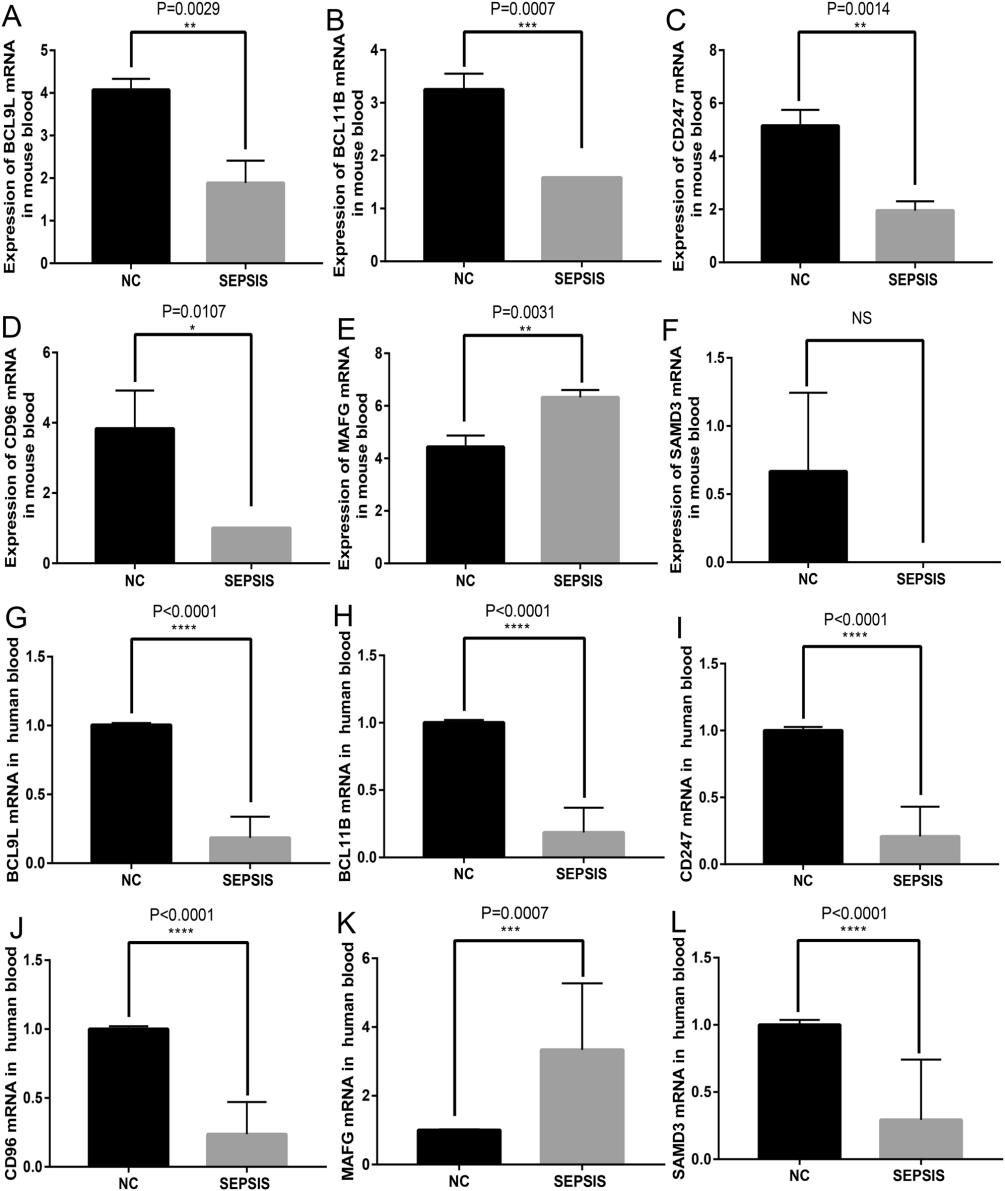
Expression tendency of key genes. (**A**–**F**) showed the statistical analysis of expression values of BCL9L, BCL11B, and CD247, CD96, MAFG, and SAMD3, in septic mouse peripheral blood measured by sequencing, respectively. The results demonstrated that these 5 genes were down-regulated in the peripheral blood of septic mice, while MAFG was up-expressed. (**G**–**L**) demonstrated the expression of BCL9L, BCL11B, and CD247, CD96, MAFG, and SAMD3 on human peripheral blood, which measured by RT-qPCR.

To further understand if the expression profiles of key genes are similar in mouse peripheral blood, septic mouse peripheral blood was subject to sequencing analysis of mRNA. It was shown that the total expression levels of BCL9L, BCL11B, CD96, SAMD3 and CD247 were down-regulated by more than twofold in the septic group (*p* < 0.01, Fig. 6A–E). The expression of MAFG was lower in sepsis and normal group (Fig. 6F). The PCR results of core genes in clinical samples verified the change trend of the expression values of these six core genes again. They were all down-regulated in sepsis group, except MAFG(Fig. 6G–L).

## Discussion

The main obstacle to the development of sepsis treatment lies in unclear its pathogenesis and clear targets relating to prognosis, making it challenging to conduct precision target therapy. In the present study, we aimed to screen potential pivotal targets affecting the prognosis of sepsis. A total of 1007 differentially expressed genes were found through RNA-seq technology and bioinformatical analysis. Three potential gene targets, including BCL9L, BCL11B, and CD247, were discovered by functional analysis combined with PPI and survival analysis. In addition, these genes were mainly expressed in T cells. They were decreased in sepsis and had a positive relation to survival, suggesting further efforts are required to confirm the potential benefits of these targets on diagnosis and treatment of sepsis.

The role of BCL9L has been wildly studied and focused on in the field of cancer. Targeting BCL9/BCL9L shows a direct anti-tumor effect, which involves anti-tumor immune responses through inhibiting Wnt and TGF-β signal transduction^17^. Dysfunction of BCL9L decreased caspase-2 level and prevented cleavage of MDM2 and BID, which contributed to aneuploidy tolerance in TP53-WT and mutated cells^18^. Upregulation of BCL9L activates Wnt/β-catenin signals and finally increases the stemness of tumor cells^19^. There is currently limited literature about the relationship between BCL9L and sepsis. We found that BCL9L was decreased in peripheral blood of septic patients and mice.

Additionally, patients with a high level of BCL9L exhibited a temporary improvement in survival rate within 28 days. GO enrichment analysis showed that BCL9L participated mainly in the BP of neutrophil activation, bacterial response, and cell killing. These findings demonstrated that BCL9L might affect the prognosis of sepsis through the above activities.

B cell chronic lymphocytic leukemia/lymphoma 11B (BCL11B) is a Kruppel-like C2H2 type zinc finger transcriptional factor relating to various malignant tumors. Recent evidence has suggested that the overexpression of BCL11B contributes to chemical resistance in malignant T cells. Inhibition of BCL11B resulted in increased cell apoptosis^20^. Decreased level of BCL11B protein is critical to the development of adult T cell leukemia and lymphoma^21^. Constitutive BCL11B mutations can result in human multiorgan system dysfunction and severe combined immunodeficiency (SCID). In addition, it causes T lymphocyte arrest and B lymphocyte dysfunction, resulting in life-threatening infection^22^. The present study found that BCL11B was correlated with a favorable sepsis prognosis and was mainly located in T cells. Sequencing analysis of mouse peripheral blood demonstrated that BCL11B decreased in sepsis compared to the control group.

CD3ζ chain (CD247) is a gene involved in T cell signal transduction, which improves the T cell antigen receptor signaling cascade^23^. CD247 plays an important role in antigen recognition and signal transduction. It is known that CD247 is related to the pathogenesis of systemic lupus erythematosus and hypertension^24,25^. We found that CD247 was lowly expressed in sepsis through sequencing analysis of human peripheral blood, followed by a lower survival rate. In combination with single-cell sequencing, it was found that CD247 is mainly expressed in T cells. These findings suggested that CD247 may participate in the pathophysiological process of sepsis.

CD96 participates in a variety of immune responses, controls immune cell infiltration, and affects the malignant characteristics of various cancer types, so it is a potential biomarker to determine patient prognosis and immune infiltration^26^. But it has not been reported in sepsis. It has been reported that SAMD3 is specifically expressed on NK cells and memory CD8 T cells during viral infection^27^. This is similar to our results. Our results suggest that CD96 and SAMD3 were located on T cells and down-regulated in sepsis group, which were positively correlated with survival rate. It is speculated that their function is helpful to the prognosis of sepsis patients. MAFG is located in single cells and upregulated in sepsis in current study, showing a negative correlation with patient survival, suggesting that MAFG is a pathogenic gene and a potential intervention target.The researchers identified astrocytes in EAE and multiple sclerosis, which are increased MAFG expression, MAFG and MAT2 α Synergistically promote DNA methylation and inhibit antioxidant and anti-inflammatory transcriptional programs^28^. This article further validates our results and provides the mechanism of action.

In this study, we integrated RNA-seq analysis of peripheral blood, 10 × single-cell sequencing technology, and sequencing of mouse peripheral blood, together with target screening closely relevant to clinical characteristics by analyzing sepsis survival data to dimensional explore the expression profile of key targets. These efforts provide important clues for further functional and mechanistic studies. There are some shortcomings in the present study. For instance, the current work is an observational study of sepsis without advanced functional verification of target genes.

## Supplementary Information

Supplementary Information.

## Data Availability

The datasets generated during the current study are available in the CNGBdb repository, (https://db.cngb.org/, project No. CNP0002611).

## References

[1] McBride MA (2020). Immune checkpoints: Novel therapeutic targets to attenuate sepsis-induced immunosuppression. Front. Immunol..

[2] Shi X, Tan S, Tan S (2021). NLRP3 inflammasome in sepsis (Review). Mol. Med. Rep..

[3] Magrone T, Jirillo E (2019). Sepsis: From historical aspects to novel vistas. Pathogenic and therapeutic considerations. Endocr. Metab. Immune Disord. Drug Targets.

[4] Kumar V (2018). T cells and their immunometabolism: A novel way to understanding sepsis immunopathogenesis and future therapeutics. Eur. J. Cell Biol..

[5] Jensen IJ (2021). Sepsis leads to lasting changes in phenotype and function of memory CD8 T cells. Elife.

[6] Alba-Patiño A (2022). Micro- and nanosensors for detecting blood pathogens and biomarkers at different points of sepsis care. Mikrochim. Acta.

[7] Opal SM, Wittebole X (2020). Biomarkers of infection and sepsis. Crit. Care Clin..

[8] Simoneau J, Dumontier S, Gosselin R, Scott MS (2021). Current RNA-seq methodology reporting limits reproducibility. Brief Bioinform..

[9] Yip SH, Sham PC, Wang J (2019). Evaluation of tools for highly variable gene discovery from single-cell RNA-seq data. Brief Bioinform..

[10] Chan JTH, Kadri S, Köllner B, Rebl A, Korytář T (2022). RNA-Seq of single fish cells - seeking out the leukocytes mediating immunity in teleost fishes. Front. Immunol..

[11] Liu, S. *et al.* Three differential expression analysis methods for rna sequencing: limma, EdgeR, DESeq2. *J. Vis. Exp.* (2021). 10.3791/62528 34605806

[12] Hill DP, Berardini TZ, Howe DG, Van Auken KM (2010). Representing ontogeny through ontology: A developmental biologist's guide to the gene ontology. Mol. Reprod. Dev..

[13] Yu G, Wang LG, Han Y, He QY (2012). clusterProfiler: An R package for comparing biological themes among gene clusters. OMICS.

[14] Scicluna BP (2015). A molecular biomarker to diagnose community-acquired pneumonia on intensive care unit admission. Am. J. Respir. Crit. Care Med..

[15] Butler A, Hoffman P, Smibert P, Papalexi E, Satija R (2018). Integrating single-cell transcriptomic data across different conditions, technologies, and species. Nat. Biotechnol..

[16] Chen M, Chen X, Hu Y, Cai X (2020). Screening of key genes related to the prognosis of mouse sepsis. Biosci. Rep..

[17] Wang X (2021). BCL9/BCL9L promotes tumorigenicity through immune-dependent and independent mechanisms in triple negative breast cancer. Oncogene.

[18] López-García C (2017). BCL9L dysfunction impairs caspase-2 expression permitting aneuploidy tolerance in colorectal cancer. Cancer Cell.

[19] Tao B (2021). Matrix stiffness promotes glioma cell stemness by activating BCL9L/Wnt/β-catenin signaling. Aging (Albany NY).

[20] Gu X, Wang Y, Zhang G, Li W, Tu P (2013). Aberrant expression of BCL11B in mycosis fungoides and its potential role in interferon-induced apoptosis. J. Dermatol..

[21] Kurosawa N (2013). Reduced level of the BCL11B protein is associated with adult T-cell leukemia/lymphoma. PLoS ONE.

[22] Punwani D (2016). Multisystem anomalies in severe combined immunodeficiency with mutant BCL11B. N. Engl. J. Med..

[23] Ye W (2019). CD247 expression is associated with differentiation and classification in ovarian cancer. Medicine (Baltimore).

[24] Li R (2012). Association of CD247 with systemic lupus erythematosus in Asian populations. Lupus.

[25] Rudemiller N, Lund H, Jacob HJ, Geurts AM, Mattson DL (2014). CD247 modulates blood pressure by altering T-lymphocyte infiltration in the kidney. Hypertension.

[26] Ye W, Luo C, Liu F, Liu Z, Chen F (2021). CD96 correlates with immune infiltration and impacts patient prognosis: A pan-cancer analysis. Front. Oncol..

[27] Peters AE, Knöpper K, Grafen A, Kastenmüller W (2022). A multifunctional mouse model to study the role of Samd3. Eur. J. Immunol..

[28] Wheeler MA, Clark IC, Tjon EC, Li Z, Zandee SEJ, Couturier CP, Watson BR, Scalisi G, Alkwai S, Rothhammer V, Rotem A, Heyman JA, Thaploo S, Sanmarco LM, Ragoussis J, Weitz DA, Petrecca K, Moffitt JR, Becher B, Antel JP, Prat A, Quintana FJ (2020). MAFG-driven astrocytes promote CNS inflammation. Nature.

